# Bioprospecting metagenomics of decaying wood: mining for new glycoside hydrolases

**DOI:** 10.1186/1754-6834-4-23

**Published:** 2011-08-04

**Authors:** Luen-Luen Li, Safiyh Taghavi, Sean M McCorkle, Yian-Biao Zhang, Michael G Blewitt, Roman Brunecky, William S Adney, Michael E Himmel, Phillip Brumm, Colleen Drinkwater, David A Mead, Susannah G Tringe, Daniel van der Lelie

**Affiliations:** 1 Brookhaven National Laboratory, Upton, NY, USA; 2 BioEnergy Science Center, Oak Ridge National Laboratory, Oak Ridge, TN, USA; 3 National Renewable Energy Laboratory, Golden, CO, USA; 4 Lucigen Corporation, Middleton, WI, USA; 5 Great Lakes Bioenergy Research Center, University of Wisconsin, Madison, WI, USA; 6 DOE Joint Genome Institute, Walnut Creek, CA, USA; 7 Center for Agriculture and Environmental Biotechnology, RTI International, Research Triangle Park, NC, USA

## Abstract

**Background:**

To efficiently deconstruct recalcitrant plant biomass to fermentable sugars in industrial processes, biocatalysts of higher performance and lower cost are required. The genetic diversity found in the metagenomes of natural microbial biomass decay communities may harbor such enzymes. Our goal was to discover and characterize new glycoside hydrolases (GHases) from microbial biomass decay communities, especially those from unknown or never previously cultivated microorganisms.

**Results:**

From the metagenome sequences of an anaerobic microbial community actively decaying poplar biomass, we identified approximately 4,000 GHase homologs. Based on homology to GHase families/activities of interest and the quality of the sequences, candidates were selected for full-length cloning and subsequent expression. As an alternative strategy, a metagenome expression library was constructed and screened for GHase activities. These combined efforts resulted in the cloning of four novel GHases that could be successfully expressed in *Escherichia coli*. Further characterization showed that two enzymes showed significant activity on *p*-nitrophenyl-α-L-arabinofuranoside, one enzyme had significant activity against *p*-nitrophenyl-β-D-glucopyranoside, and one enzyme showed significant activity against *p*-nitrophenyl-β-D-xylopyranoside. Enzymes were also tested in the presence of ionic liquids.

**Conclusions:**

Metagenomics provides a good resource for mining novel biomass degrading enzymes and for screening of cellulolytic enzyme activities. The four GHases that were cloned may have potential application for deconstruction of biomass pretreated with ionic liquids, as they remain active in the presence of up to 20% ionic liquid (except for 1-ethyl-3-methylimidazolium diethyl phosphate). Alternatively, ionic liquids might be used to immobilize or stabilize these enzymes for minimal solvent processing of biomass.

## Background

In recent years, and in the face of depletion of fossil fuel resources and a growing global environmental awareness, biofuels have attracted more interest as an alternative, renewable source of energy. Plant biomass has long been recognized as a potential sustainable source of mixed sugars for biofuels production via fermentation. However, in order to develop cost-effective processes for converting biomass to fuels and chemicals several technical barriers related to biomass recalcitrance, such as attainment of minimal biomass pretreatments matched to active enzymes, still need to be overcome [1]. In nature, cellulosic biomass is decomposed by complex and efficient microbial processes. Various microorganisms produce cellulolytic enzymes that function synergistically to decompose plant biomass [2-4]. These environments contain microbial communities that can efficiently decompose natural plant biomass; they include the animal rumen [5-8], digestive tracks of termites [9-11] and wood boring insects [12], and decomposed biomass [13-15]. Many of these systems have proved to be attractive sources for exploring novel plant biomass degrading organisms and enzymes.

Estimates suggest that approximately 4-6 × 10^30 ^prokaryotes inhabit the Earth [16] and constitute the world's major reserve of genetic diversity. However, about 95 to 99.9% of microorganisms have not been cultured by standard laboratory techniques [17,18]. In order to bypass the limitation of cultivation-based methodologies, metagenomic approaches became a powerful tool to directly study the diversity of genes within microbial communities, analyze their biochemical activities, and prospect novel biocatalysts from environmental samples [19-22]. Advances in high-throughput sequencing technologies have provided tools with lower cost and facilitated the progression of metagenome projects.

Recently, we sequenced the metagenome of a mesophilc, anaerobic microbial community that actively decays poplar woodchips (van der Lelie, Taghavi, McCorkle, Li, Monteleone, Himmel, Donohoe, Ding, Adney, and Tringe unpublished results). The metagenomic DNA was cloned into plasmid and fosmid libraries for paired-end Sanger sequencing, and later directly sequenced by 454 pyrosequencing. In addition, selected fosmids containing putative glycoside hydrolases (GHases) were pooled and sequenced using 454 pyrosequencing. Approximately 675 Mb of sequence was generated and after assembly, resulted in 44,600 contigs and 1.42 M singletons totaling 382 Mb.

To mine this metagenome for new plant biomass degrading enzymes, tiled blastx was used to search against the CAZy database and approximately 4,000 glycoside hydrolase homologs were identified. A metagenomic shotgun expression library was also constructed and screened for GHase activities. The most active enzymes, identified by hydrolysis of chromophoric sugar aglycones, were selected for further investigation; including gene cloning, protein expression, and preliminary enzyme characterization. Activities of some enzymes were tested in the presence of ionic liquids, an emerging technology for biomass pretreatment as well as a new approach to increase enzyme stability and activity during minimal solvent processing [23].

## Results

### Mining for glycoside hydrolases

**Figure 1:**
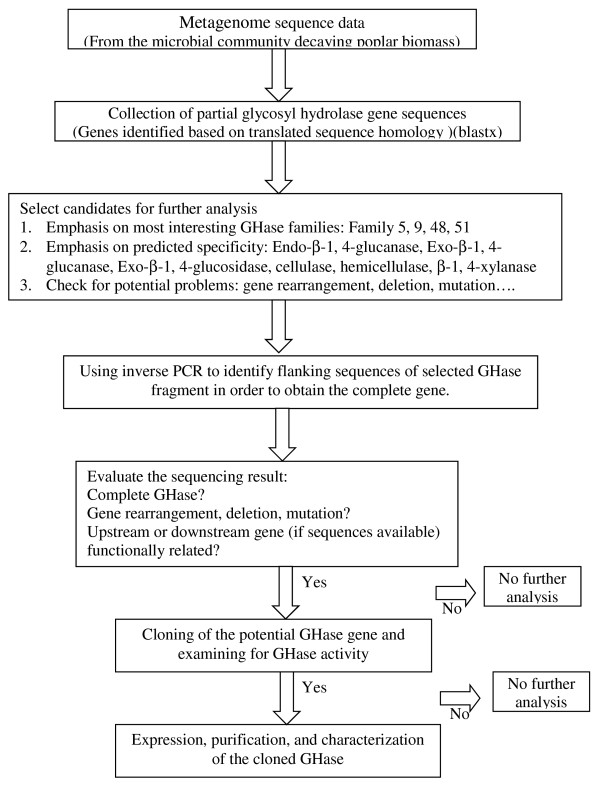
**Cloning strategy of this study**.

In a previous study (van der Lelie *et al*., unpublished results); the metagenome of a microbial community that actively decays poplar wood chips was sequenced. Since this enzyme-mining project was started before finishing the primary metagenome sequencing and assembly work, enzyme candidates for this study were selected from the sequencing data described below. The initial results from this sequencing project were generated from paired-end Sanger sequencing of a short-insert metagenome library (about 6 Mbp). We also successfully constructed a metagenome fosmid library with an average insert size 40 kb. After initial pair-end sequencing, 454-GS-FLX Titanium sequencing of 45 pooled fosmids were selected based on sequence homology with putative GHases. This work generated an additional 1.8 Mbp (that is, 7.8 Mbp total). As previously discussed by Allgaier *et al*. [13] and Li *et al*. [21], full-length genes are desirable for enzyme characterization, but difficult to obtain from highly fragmented metagenome sequence data. Therefore, candidate genes were selected based on the following criteria: (1) homology to GHase families/activities encoding key enzymes for efficient decay of recalcitrant plant cell wall polymers, especially GHase families 5, 9, 48, and 51; and (2) quality of sequences, where each candidate gene was compared to the length and the percentage of homology to its closest homologs and then examined for potential gene rearrangements, disruptions, deletions, or mutations. Candidates who had homology with enzyme families of interest and no obvious sequence rearrangements were selected for further analysis. A schematic representation of the cloning strategy is shown in Figure 1.

### Single nucleotide polymorphism of putative glycoside hydrolases

Full-length open reading frames (ORFs) and flanking sequences were obtained using inverse PCR and DNA walking. From the first set of Sanger-based sequences, nine candidate GHases were initially selected. However, after sequence analysis (Figure 1), only three candidates showed the correct ORF and homology to merit further characterization. Similarly, from the 454-based fosmid sequences, ten candidate genes were selected, but only five were selected for further experiments. The descriptions of the selected GHase candidates are listed in Table 1.

**Table 1:** Descriptions of selected glycosyl hydrolase candidates

**Contig no./clone no**.	Homolog	**Genbank accession no**.
2412	GH10 endo-1,4-beta-xylanase [Paenibacillus barcinonensis] GH10 intra-cellular xylanase [uncultured bacterium] GH10 exo-beta-1,4-xylanase [Aeromonas punctata]	JF422034
5950	GH9 endochitinase [Vibrio parahaemolyticus AQ3810] GH9 endoglucanase-related protein [Vibrio alginolyticus 12G01] GH9 glucosamine-link cellobiase [Photobacterium damselae subsp. damselae CIP 102761]	JF422035
889	GH9 glycosyl hydrolase family 9 [Listeria monocytogenes str. 1/2a F6854, 4b F2365, 4b H7858] GH9 glycosyl hydrolase, family 9 protein [Bacteroides sp. D20] GH9 glycosyl hydrolase, family 9 protein [Clostridium hathewayi DSM 13479 ]	JF422036
No. 4	GH51 alpha-L-arabinofuranosidase; Glycosyl hydrolase family 51 [Flavobacterium johnsoniae UW101] GH51 glycosyl hydrolase family 51, candidate alpha-L-arabinofuranosidase [Parabacteroides distasonis ATCC 8503] GH51 alpha-L-arabinofuranosidase A precursor [Bacteroides thetaiotaomicron VPI-5482]	JF422030
No. 5	GH51 glycosyl hydrolase family 51 [Bacteroides vulgatus ATCC 8482] GH51 alpha-L-arabinofuranosidase A precursor [Bacteroides thetaiotaomicron VPI-5482] GH51 alpha-*N*-arabinofuranosidase [Opitutus terrae PB90-1]	JF422031
No. 6	GH51 alpha-L-arabinofuranosidase; Glycosyl hydrolase family 51 [Flavobacterium johnsoniae UW101] GH51 alpha-L-arabinofuranosidase [Gramella forsetii KT0803] GH51 alpha-L-arabinofuranosidase domain protein [Clostridium cellulolyticum H10]	JF422025
No. 8	GH9 cellulase [Solibacter usitatus Ellin6076] GH9 glycosyl hydrolase, family 9 [Acidobacterium capsulatum ATCC 51196] GH9 glycosyl hydrolase family 9 [Clostridium cellulolyticum H10]	JF422032
No. 9	S-layer domain protein [Paenibacillus sp. JDR-2] Sugar-binding domain protein [Clostridium cellulolyticum H10]	JF422033

**Figure 2:**
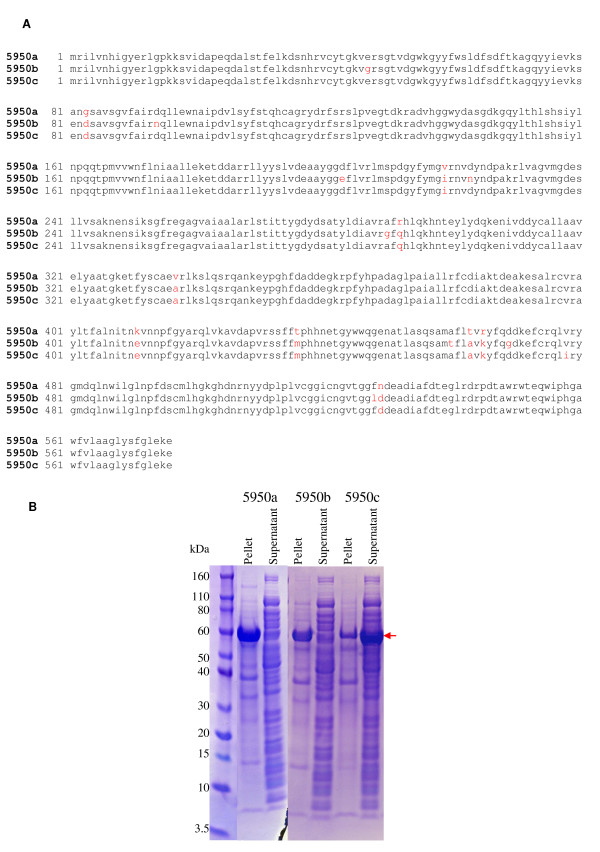
**Sequence polymorphism in contig 5950**. **(a) **Amino acid sequences of three clones with single nucleotide polymorphisms (SNPs) in contig 5950 (5950a, b, and c) were shown. Differences between these three clones are indicated in red. **(b) **SDS-PAGE analysis of SNP clones. After treating with sonication, the pellet fraction and the supernatant fraction of each clone were analyzed. As indicated in a red arrow, the 5950c clone appeared to have more expressed protein present in the supernatant.

During the process of DNA walking and sequencing, our sequencing results suggested the possibility of intragene single nucleotide polymorphism (SNP). These few variations were not generated by PCR/sequencing errors and were also observed during metagenome sequencing. As an example, we cloned three variations of candidate gene 5950 (sequences showed in Figure 2a) and expressed them in *Escherichia coli*. As shown in Figure 2b two clones, 5950a and 5950b, produced mostly insoluble protein that appeared in the pellet fraction. Interestingly, clone 5950c produced mostly soluble protein that appeared in the supernatant fraction (seven independent colonies of clone 5950c were tested, all of them predominantly producing the protein in the soluble fraction). This result points to a relationship between sequence polymorphisms and protein properties, such as protein solubility.

### Cloning, expression, and characterization of candidate glycoside hydrolases

**Figure 3:**
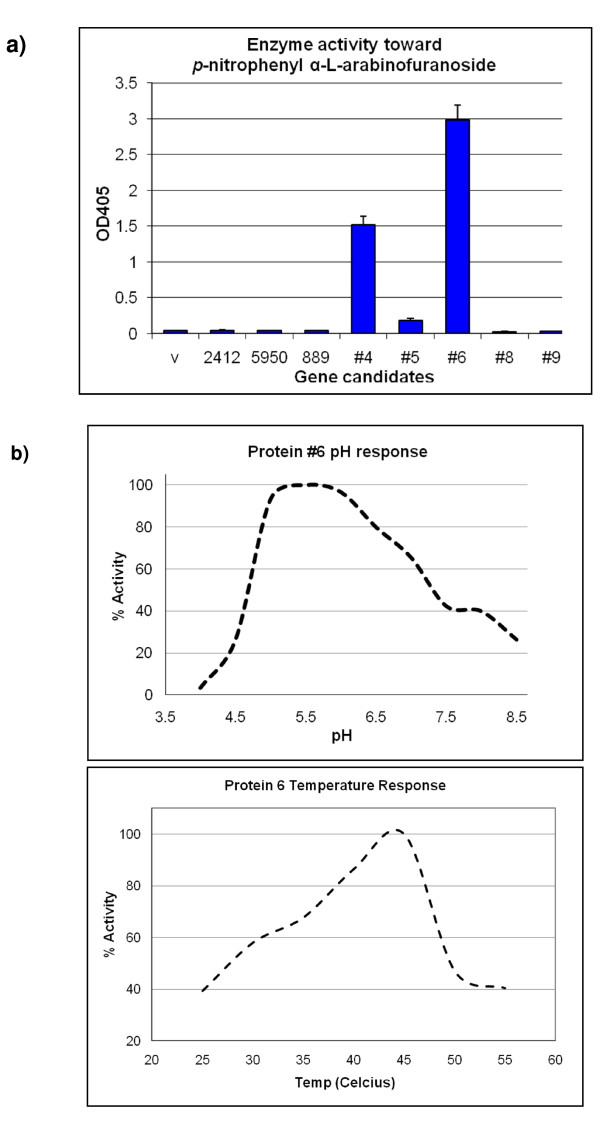
**Enzyme characterization of candidate glycosyl hydrolases (eight clones directly from metagenomic DNA)**. **(a) **Activity of cell lysates toward *p*-nitrophenyl α-L-arabinofuranoside. **(b) **Optimal pH and temperature for no. 6 enzyme reaction.

Initially, eight full-length candidate genes were cloned into the T7 expression vector pET28a (Novagen, Gibbstown, NJ, USA) with a polyhistidine tag sequence (His-tag) at the N-terminus. In order to explore the possibility of better protein expression and solubility, a second set of clones were constructed with a C-terminal His-tag and deletion of probable signal peptide sequences. All constructs were expressed in *E. coli *and cell lysates were examined with SDS-PAGE. To examine their enzyme activities, cell lysates of the eight candidate genes expressing clones and the control (*E. coli *with vector pET28a) were tested against the following substrates: *p*-nitrophenyl β-D-cellobioside, *p*-nitrophenyl β-D-glucopyranoside, *p*-nitrophenyl β-D-lactopyranoside, *p*-nitrophenyl β-D-galactopyranoside, *p*-nitrophenyl β-D-xylopyranoside, and *p*-nitrophenyl α-L-arabinofuranoside. With a 1 h enzyme reaction time, clones no. 4 and no. 6 showed significant enzyme activity toward *p*-nitrophenyl α-L-arabinofuranoside. Clone no. 5 also had a lower activity to *p*-nitrophenyl α-L-arabinofuranoside (Figure 3a). No enzyme activity was observed for the other clones, including 5950. Therefore, clone no. 4, no. 5, and no. 6 were further investigated with larger scale protein expression and purification as described in the Methods section. Unfortunately, shortly after elution, the protein of clone no. 4 precipitated and no enzyme activity could be detected. Although the protein of clone no. 5 stayed soluble after dialysis and protein concentration, no enzyme activity could be detected from the purified protein. The clone no. 6 protein remained soluble and active throughout the purification process. Therefore, the purified clone no. 6 protein was further investigated for the optimal enzyme reaction pH and temperature. As shown in Figure 3b, it had optimal activity toward *p*-nitrophenyl α-L-arabinofuranoside at pH 5 to 6, 45°C.

### Mining glycoside hydrolases from a metagenomic expression library

**Figure 4:**
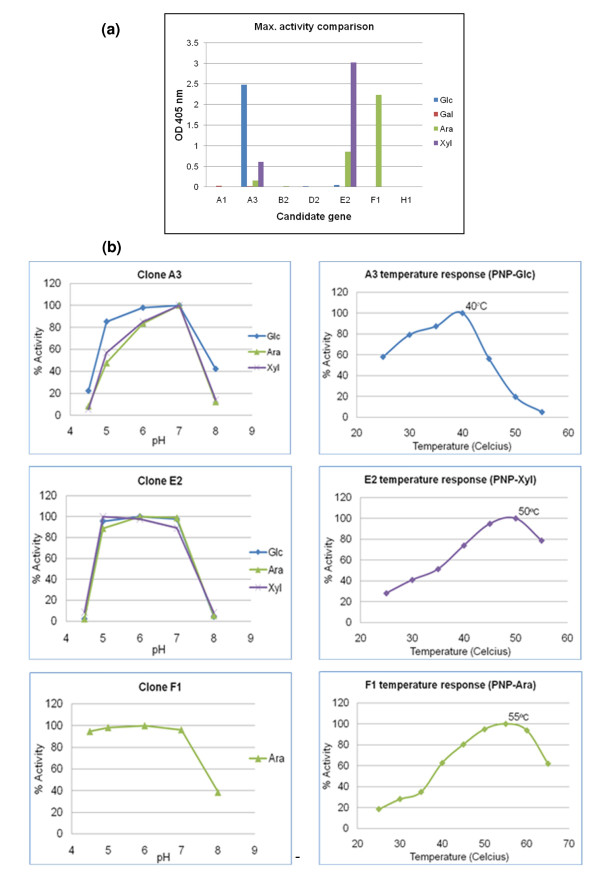
**Enzyme characterization of candidate glycosyl hydrolases (four clones from the metagenomic expression library)**. **(a) **Enzyme activities against one or more substrates: *p*-nitrophenyl β-D-glucopyranoside, *p*-nitrophenyl β-D-galactopyranoside, *p*-nitrophenyl β-D-xylopyranoside, and *p*-nitrophenyl α-L-arabinofuranoside. **(b) **Optimal pH and temperature for enzyme reaction of protein A3, E2, and F1.

Function-based screening of metagenomic expression libraries is another approach to mining glycoside hydrolases from metagenomes. Using this approach, some previously unknown genes that do not share homology with known GHases can be discovered and accessed. Furthermore, the sequences and enzyme activities are functionally guaranteed. In order to mine for new glycoside hydrolases from the microbial community decaying poplar wood chips, a random shotgun metagenomic expression library was constructed. Initial screening of the expression library revealed 45 positive candidate clones using azurine-crosslinked polysaccharides (AZCL-HE-cellulose, AZCL-arabinoxylan, and AZCL-barley β-glucan) and fluorogenic substrates (5-bromo-6-chloro-3-indolyl-β-D-glucopyranoside, 4-methylumbelliferyl-β-D-xylopyranoside, and 4-methylumbelliferyl-β-D-cellobiopyranoside) as substrates. These 45 clones were further screened by using chromogenic substrates *p*-nitrophenyl-cellobioside, *p*-nitrophenyl-lactopyranoside, *p*-nitrophenyl-β-galactopyranoside, *p*-nitrophenyl-xylopyranoside, *p*-nitrophenyl-arabinofuranoside, and *p*-nitrophenyl-glucopyranoside as substrates. All clones showed activity toward *p*-nitrophenyl-β-galactopyranoside, resulting from a background β-galactosidase activity by the *E. coli *host. Clones A1, F1, H1, B2, D2, E2, and A3 also showed activity toward *p*-nitrophenyl-cellobioside, *p*-nitrophenyl-lactopyranoside, *p*-nitrophenyl-xylopyranoside, *p*-nitrophenyl-arabinofuranoside, or *p*-nitrophenyl-glucopyranoside. This result implies that these clones may have activities toward hemicellulose and/or cellulose. Therefore, we further performed DNA sequencing and analyzed the full-length inserts of these seven clones. For all clones, putative ORFs were identified and blastx analysis was used to identify homologs to genes with known glycoside hydrolase activity (see Table 2). The result of the sequence analysis suggested that these putative glycoside hydrolases might not necessarily be transcripted from the T7 promoter of the library vector, as some of the putative GHase encoding ORFs were oriented in the opposite direction as the orientation of transcription from this promoter. Therefore, and for the purpose of easier protein purification, we reconstructed each of these putative glycoside hydrolases as a His-tag fusion protein in pET28a. Whole cell lysates of these constructs were subsequently tested for protein expression and putative glycoside hydrolases activities. Potential enzyme activities were screened on *p*-nitrophenyl β-D-cellobioside, *p*-nitrophenyl β-D-glucopyranoside, *p*-nitrophenyl β-D-lactopyranoside, *p*-nitrophenyl β-D-galactopyranoside, *p*-nitrophenyl β-D-xylopyranoside, and *p*-nitrophenyl α-L-arabinofuranoside under various conditions of pH (pH 4.5 to 8) and temperature (25 to 55°C). For all seven constructs, no obvious activity was seen toward *p*-nitrophenyl β-D-cellobioside and *p*-nitrophenyl β-D-lactopyranoside. Enzyme activities against one or more substrates were, however, observed for clones A3, E2, and F1 (Figure 4a). Clone A3 showed activities against *p*-nitrophenyl β-D-glucopyranoside, *p*-nitrophenyl β-D-xylopyranoside, and *p*-nitrophenyl α-L-arabinofuranoside; clone E2 against *p*-nitrophenyl β-D-xylopyranoside, and *p*-nitrophenyl α-L-arabinofuranoside. Clone F1 was active on *p*-nitrophenyl α-L-arabinofuranoside. In Figure 4b, the pH and temperature dependencies are shown for the clone A3, E2, and F1 proteins. Protein A3 has optimal activity against *p*-nitrophenyl β-D-glucopyranoside at pH 6-7, 40°C; protein E2 has optimal activity against *p*-nitrophenyl β-D-xylopyranoside at pH 5-6, 50°C; and protein F1 has optimal activity against *p*-nitrophenyl α-L-arabinofuranoside at pH 5-6, 55°C.

**Table 2:** Blastx results of full-length positive clone inserts

Clone	Homologs	**Genbank accession no**.
A1	Ribokinase-like domain-containing protein [Clostridium beijerinckii NCIMB 8052] Sugar kinases, ribokinase family [Ruminococcus sp. SR1/5] PfkB domain protein [Olsenella uli DSM 7084]	JF422026
F1	Alpha-L-arabinofuranosidase [Clostridium stercorarium] Alpha-L-arabinofuranosidase domain protein [Thermoanaerobacterium thermosaccharolyticum DSM 571] Arabinofuranosidase [Geobacillus stearothermophilus]	JF422024
H1	2-Methyleneglutarate mutase [Natranaerobius thermophilus JW/NM-WN-LF] 2-Methyleneglutarate mutase [Eubacterium barkeri] Hypothetical protein BACCAP_02289 [Bacteroides capillosus ATCC 29799]	JF422029
B2	Beta-galactosidase [Clostridium hathewayi DSM 13479] Beta-glucosidase [Sorangium cellulosum 'So ce 56'] Beta-glucosidase [Acaryochloris marina MBIC11017]	JF422027
D2	Beta-xylosidase, putative, xyl39A [Cellvibrio japonicus Ueda107] Candidate beta-xylosidase; Glycoside hydrolase family 39 [Flavobacterium johnsoniae UW101] Glycoside hydrolase family 39 domain protein [Teredinibacter turnerae T7901]	JF422028
E2	Beta-xylosidase B [Clostridium stercorarium] Glycoside hydrolase family 3 domain protein [Clostridium papyrosolvens DSM 2782] Glycoside hydrolase family 3 domain protein [Clostridium cellulolyticum H10]	JF422023
A3	*N*-acetyl-beta-glucosaminidase [Cellulomonas fimi] Glycosyl hydrolase, family 3 [Clostridium hathewayi DSM 13479] Beta-glucosidase-related glycosidases [Roseburia intestinalis XB6B4]	JF422022

### Enzyme purification and activity quantification

**Figure 5:**
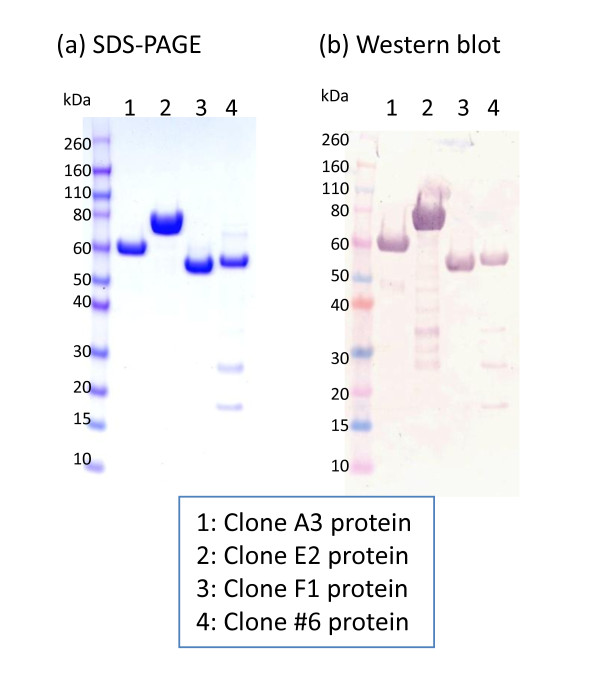
**Examination of purified proteins**. Four purified proteins were examined by using SDS-PAGE **(a) **and western blot with the anti-His-tag antibody **(b)**.

The four candidate clones that showed significant enzyme activities (clone no. 6, A3, E2, and F1) were cultured and expressed proteins were purified as described in the Methods section. As is shown in Figure 5, these four purified proteins were examined by using SDS-PAGE (a) and western blot with the anti-His-tag antibody (b). Quantification of the enzyme activity was also estimated using a *p*-nitrophenol standard curve. Approximate enzyme activity of these proteins were: 1 μg clone no. 6 protein can release about 7.14 nmol of *p*-nitrophenol from *p*-nitrophenyl α-L-arabinofuranoside per min, 1 μg clone A3 protein can release about 0.96 nmol of *p*-nitrophenol from *p*-nitrophenyl β-D-glucopyranoside per min, 1 μg clone E2 protein can release about 6.19 nmol of *p*-nitrophenol from *p*-nitrophenyl β-D-xylopyranoside per min, and 1 μg clone F1 protein can release about 21.12 nmol of *p*-nitrophenol from *p*-nitrophenyl α-L-arabinofuranoside per min.

### Enzyme properties: the tolerance for ionic liquids

In order to make the lignocellulosic biomass more accessible by hydrolytic enzymes and release more sugars, pretreatments of the biomass such as thermochemical pretreatment or acid treatment are usually applied before the step of enzyme hydrolysis [24]. Furthermore, the subsequent hydrolysis of the biomass into fermentable sugars requires enzymes that remain active under conditions of high substrate loading and minimal solvent. The discovery of cellulose-dissolving ionic liquids in recent years suggests a new and 'greener' direction for processing of lignocellulosic materials [25-27] and to improve enzyme stability and activity under minimal solvent processing conditions [23]. However, there are concerns regarding retention of enzyme activities in the presence of ionic liquids [28]. Currently, available industrial processes for ionic liquid treatment will leave around 10% (v/v) residual ionic liquid. We therefore tested enzyme activities in various concentrations of ionic liquids.

**Figure 6:**
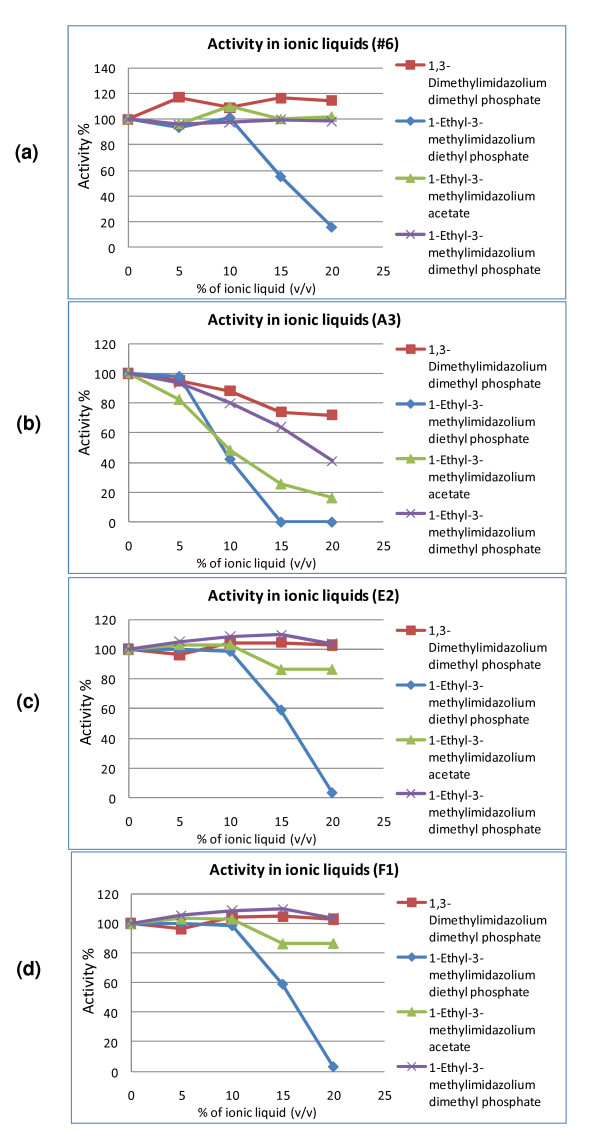
**Enzyme activities in the presence of ionic liquids**. **(a) **Protein no. 6 activity toward *p*-nitrophenyl α-L-arabinofuranoside in the presence of ionic liquids. **(b) **Protein A3 activity toward *p*-nitrophenyl β-D-glucopyranoside in the presence of ionic liquids. **(c) **Protein E2 activity toward *p*-nitrophenyl β-D-xylopyranoside in the presence of ionic liquids. **(d) **Protein F1 activity toward *p*-nitrophenyl α-L-arabinofuranoside in the presence of ionic liquids.

The effects of four ionic liquids, 1,3-dimethylimidazolium dimethyl phosphate, 1-ethyl-3-methylimidazolium diethyl phosphate, 1-ethyl-3-methylimidazolium acetate, and 1-ethyl-3-methylimidazolium dimethyl phosphate, on enzyme activity are shown in Figure 6. Enzyme activities in the presence of ionic liquids were compared with activities in buffer alone and these controls were set as 100%. Generally, no dramatic change in enzyme activity was observed when the concentration of ionic liquid was below 5%. All four enzymes appeared to be less tolerant to higher concentrations of 1-ethyl-3-methylimidazolium diethyl phosphate and protein A3 also appeared to be less tolerant to all four ionic liquids as compared with the other three proteins. The activity of the clone no. 6 protein went up about 20% in the presence of 1,3-dimethylimidazolium dimethyl phosphate (120% activity). This result suggested that the 100% removal of ionic liquid from biomass after treatment may be not necessary if enzymes that will be used in the saccharification process can tolerate ionic liquids. It also shows that 1,3-dimethylimidazolium dimethyl phosphate can be used to improve the reaction rates of the clone no. 6 protein.

## Discussion

Since the publication in 1991 by Schmidt and coworkers that described the concept of a metagenome [29], it has become a very powerful tool for the study of biodiversity in the environment and to explore novel enzymes for bioindustrial and biomedical applications. In this study, we have mined new glycoside hydrolases from the metagenome of a poplar biomass-decaying microbial community using both a sequence-based approach and a function-based approach. In this sequence-based approach, all eight of the initial sequence-confirmed ORF candidates show protein expression in the *E. coli *host. Six ORF candidates have variable amounts of expressed proteins found in the soluble fraction and the remaining three ORF candidates showed detectable enzyme activity in their cell lysates. However, only one candidate retains its protein solubility and good enzyme activity after the protein purification process. However, with the function-based library screening approach using the 45 positive clone library, of the clones picked up by the initial screening, 7 of them contain homologous glycoside hydrolase coding sequences in the insert sequence and show enzyme activity in the cell lysates. The remaining three clones still retain their protein solubility and good enzyme activity after the protein expression and purification processes.

Blastx comparison showed that the amino acid sequence homologies of the glycoside hydrolases isolated and characterized in this study ranged from approximately 50% to 70% when compared to that of their closest homologs (50% homology for the proteins from clones, A3 and F1, 60% for the clone E1 protein, and 68% for the clone no. 6 protein, respectively). Therefore, our results show that truly new and active glycoside hydrolases can be obtained from the poplar biomass decaying metagenome by using both a sequence-based search and a function-based screening.

During the process of direct DNA cloning from the metagenomic DNA, the possibility of intragene SNP was observed. Our results have suggested the possible relationship between sequence polymorphisms and protein properties, such as protein solubility (clone 5950c in Figure 2 as an example). Although further studies of protein 5950c was not continued, because no significant enzyme activity could be observed, SNP may still serve as a resource for different protein properties when cloning from environmental samples, such as metagenomic DNA.

According to our results, function-based screening seems to have a better chance to discover active enzymes than the sequence-based searches. As discussed in a previous review [21], the advantage of directly screening for enzymatic activities from metagenomic libraries is that enzyme activities are functionally guaranteed. Indeed, this approach did bring us more functional enzymes. However, the limitation to this approach is that the clone must contain the complete gene sequence, or even a gene cluster. Sequence-based screening methods, however, rely on known conserved sequences and experiments are the only way to ensure enzyme activities. Yet, this method can disclose target genes regardless of the completeness of the target gene's sequence. Currently, most of limitations of sequence-based searches are technical issues, for instance, the quality of sequencing reads (length, error rates) and accuracy of sequence assembly. In fact, among the 20 initial selections of candidate fragments, 3 of them were eliminated due to sequencing/assembly errors present in the metagenomics data. Despite this, with the development and improvement of new sequencing technology and bioinfomatics tools, we believe these limitations will be solved soon.

In this study, we have successfully cloned four new glycoside hydrolases from the metagenome of a decaying poplar biomass microbial community. Two enzymes (no. 6, F1) have significant activity on the substrate *p*-nitrophenyl α-L-arabinofuranoside, one enzyme (A3) has significant activity on the substrate *p*-nitrophenyl β-D-glucopyranoside, and one enzyme (E2) has significant activity on the substrate *p*-nitrophenyl β-D-xylopyranoside. These four cloned enzymes could be interesting not only because they can be expressed in *E. coli *and still retain significant activity after protein purification process, but they also have a certain level of tolerance to the four ionic liquids that we tested. Enzyme activities were evaluated for ionic liquid concentrations of up to 20%; no higher concentrations were tested since these products are very expensive and in addition after their removal the concentration is never that high. Three enzymes remained at nearly 100% activity in the presence of up to 20% of 1,3-dimethylimidazolium dimethyl phosphate, 1-ethyl-3-methylimidazolium acetate, and 1-ethyl-3-methylimidazolium dimethyl phosphate. However, all four enzymes appeared to be less tolerant to higher concentrations of 1-ethyl-3-methylimidazolium diethyl phosphate, while protein A3 also appeared to be less tolerant to all four ionic liquids as compared with the other three proteins. The activity of clone no. 6 went up about 20% in the presence of 1,3-dimethylimidazolium dimethyl phosphate, probably as a result from changes in surface properties due to the presence of this ionic liquid (120% activity, see Figure 6). This opens the possibility for improved hydrolysis of biomass using this combination of enzyme and ionic liquid under processing conditions characterized by high biomass loadings and minimal solvent concentrations. Furthermore, these enzymes may be useful for processing ionic liquid-treated biomass without the need of intensive washes to dilute ionic liquid residues, thus helping to reduce the use of water after the ionic liquid treatment. In a laboratory setting, repeated washing of biomass to rinse off remaining ionic liquids can be easily achieved without considering the consumption of water. However, in an industrial setting, the cost and restrictions of water usage need to be seriously taken into consideration. Currently the available industrial processes for recovering ionic liquid from treated biomass will leave around 10% (v/v) residual ionic liquid. Therefore, it is a benefit if the activity of an enzyme is not negatively affected by the presence of 10% ionic liquid.

Two of the enzymes studied in detail, E2 and F1, show a temperate activity profiles indicating strong retention of activity at elevated temperatures (that is, 40% to 50% retention of activity at 60°C). These enzymes would be good candidates to use in many mildly thermophilic enzyme cocktails, including those from *Thermobifida fusca *and *Clostridium thermocellum*. Indeed, all three clones studied (no. 6, A3, E2, and F1) could be useful in both fungal and bacterial enzyme mixtures considering the broad pH range of activity retention (see Figure 4b). We also note that these four enzymes still have the His-tags attached. Therefore, these four enzymes have the potential to be easily recovered after the treatment slurry and could be recycled. There is also the potential to use a His-tag to immobilize these enzymes and then apply them in a continuous reaction systems, eventually combined with the application of ionic liquids [23]. Further studies will be necessary to optimize conditions for specific reactions and perhaps improve the wild type enzyme performance. For instance, the His-tag may be replaced with a more suitable tag for the immobilization purpose, because we already know the His-tag in this position did not disrupt the protein folding and enzyme activity.

By using both the sequence-based search and the function-based screening, we have identified 15 promising clones coding enzymes likely to be critical for bacterial degradation of biomass. Four of these clones provided new, stable and active glycoside hydrolases from the metagenome of a decaying poplar. Some of the 15 clones code for enzymes that are of the monosaccharide aglycone cleaving type; that is, clones no. 4, no. 5, no. 6 and F1 are consistent with the GH51 family which contains enzymes (EC3.2.1.55) that hydrolyze α-L-arabinofuranosides from the arabinogalactan backbone of tension wood in hard woods. B2 is consistent with the GH1 family, which contains enzymes (EC 3.2.1.21) that hydrolyze cellobiose to glucose; as well as other disaccharides to monosaccharide units (EC 3.2.1.23 β-D-galactosidase and 3.2.1.25 β-D-mannosidase); β-D-glucuronidases (EC 3.2.1.31) are also found in this GH family and these enzymes may be required to hydrolyze linkages in the tension wood of hard woods. D2 and E2 (A3) are consistent with the GH39 and GH3 families, respectively, which contain enzymes (EC 3.2.1.37) that hydrolyze xylobiose to xylose and/or remove successive D-xylose residues from the non-reducing termini of xylan in hard woods. The enzymes consistent with clones no. 5950, no. 889, and no. 8 are found in the GH9 family of enzymes (EC 3.2.1.4) that hydrolyze the insoluble polysaccharide, cellulose, to cellobiose and glucose. The enzymes consistent with the no. 2412 clone (EC 3.2.1.8) hydrolyze the branched polysaccharide, xylan, to xylose and xylooligomers. These polymer-degrading enzymes are all expected for the bacterial saccharification of hard woods. The enzymes consistent with clone no. 9 are found in cellulosomal enzymes systems, where S-layer proteins in the bacterial cell wall are tethered to linker peptide bound cellulosomes. The enzymes suggested by sequence homology for clones A1 and H1 would not be expected to directly play a role in the digestion of biomass.

In this study, azurine crosslinked polysaccharides and colorimetric substrates were used to evaluate glycoside hydrolase activities. These standardized substrates were used to permit direct comparison within the context of this study; where only small quantities of enzymes were available. In future work, selected enzymes could be prepared at large scale for hydrolysis testing of pretreated biomass feedstocks under conditions relevant to the industrial saccharification process [30]. Therefore, future studies will include non-artificial substrates for enzyme activity testing.

## Conclusions

We have demonstrated that the metagenome method can be a good resource to explore and prospect new functional enzymes for biomass deconstruction and biofuels production. Importantly, analysis of the GHases from this polar decaying wood pile revealed the production of cell wall degrading enzymes entirely consistent with the specific glycosidic linkages expected for the bacterial deconstruction of hard woods. The four GHases that were cloned may have potential application for deconstruction of biomass pretreated with ionic liquids, as they remain active in the presence of up to 20% ionic liquid, except when 1-ethyl-3-methylimidazolium diethyl phosphate is present. Alternatively, ionic liquids might be used to immobilize or stabilize these enzymes for minimal solvent processing of biomass.

## Methods

### Metagenome DNA, data mining and target genes selection

This work concentrated on the microbial community decaying poplar biomass under anaerobic conditions. A total of 1.8 kg non-sterile yellow poplar sawdust, with particles ranging in sizes between 1 mm^3 ^to 0.3 cm^3^, was taken from the inside of a 1 m^3 ^pile and placed in a white, plastic, 10 l bucket. The biomass was humidified with 5 l of 10 mM MgSO_4 _solution and the bucket was closed with an airtight plastic cover. This resulted in the creation of a gradient ranging from micro aerobic at the top to anaerobic at the bottom of the biomass. After 12 months of incubation in the dark at 30°C, 500 g biomass and 500 ml liquid were collected from the anaerobic zone at the bottom of the bucket and used for DNA isolation. Metagenome sequencing and data analysis were described in a separated publication (van der Lelie *et al*., unpublished results). The metagenome data can be publically accessed via the IMG/M website at http://img.jgi.doe.gov/cgi-bin/m/main.cgi?section=TaxonDetail&taxon_oid=2010388001 To prospect for genes encoding glycoside hydrolases in the decaying poplar biomass microbial community, the tiled blastx searches was performed against the CAZy database http://www.cazy.org/ (filtered with E-value of 1^-10 ^or better). Approximately 4,000 putative glycoside hydrolase homologs were identified. From these homologs, candidate genes were selected for further investigation based on following categories. (1) Enzyme functions of interests. GHase families that represent key enzymes for the most efficient decomposition of plant cell wall recalcitrants: cellulase (GH5, 6, 8, 9, 48); hemicellulase (GH 8, 10, 11, 12, 26, 28, 53, 74); debranch enzyme (GH51, 54, 62, 67, 78, 74). (2) The quality of sequences, including gene length and homology, and exclude genes with potential gene rearrangements, disruptions, and deletions. A scheme of the cloning strategy is showed in Figure 1, and descriptions of selected glycoside hydrolase candidates are listed in Table 1.

### Construction of random shotgun metagenomic expression library

Purified metagenomic DNA (approximately 1 μg) was fragmented by hydrodynamic shearing (HydroShear apparatus, Digilab, Holliston, MA, USA) to generate fragments of 2-4 kb. The fragments were end-repaired enzymatically (DNATerminator kit, Lucigen, Middleton, WI, USA) purified on an agarose gel, and ligated to pETite, a small T7 promoter vector (Lucigen). The recombinant plasmids were then used to transform electrocompetent HI-Control BL21(DE3) cells (Lucigen), which contain a single-copy BAC plasmid harboring a specially engineered version of the *lacI^q1 ^*repressor allele. A total of 20,000 clones were picked and screened for carbohydrase activity using 4 substrates simultaneously. Clones were grown overnight in a 1 ml × 96 deep well plate using Overnight Express medium (EMD, Gibbstown, NJ, USA), pelleted by centrifugation, and lysed using CelLytic B Reagent (Sigma-Aldrich, St Louis, MO, USA). Enzyme assays for cellulase, xylanase, β-xylosidase, and β-glucosidase were performed simultaneously by mixing substrate containing 0.2% AZCL-arabinoxylan, 0.2% AZCL-HE cellulose, 0.02% methylumbelliferyl-β-D-xylopyranoside and 0.002% magenta-glucoside in 50 mM acetate buffer, pH 5.8 and adding to lysated pellets. Reactions were incubated overnight at 37°C with shaking. Plates were assayed by centrifugation and transferring aliquots of the supernatant to 96 well plates for fluorescence and absorbance measurements. A total of 45 carbohydrase active clones were identified and the DNA inserts sequenced using conventional Sanger chemistry sequencing.

### Cloning the full-length ORF of glycoside hydrolase gene directly from metagemonic DNA

To obtain flanking sequences of candidate gene fragments (in order to reconstruct the full-length ORF of each candidate), inverse PCR and DNA walking were performed. For inverse PCR, purified metagemone DNA was partially digested with restriction endonuclease *Bam*HI or *Eco*RI and subsequently diluted and treated with T4 DNA ligase. Two sets of primers that are specific to each candidate gene were used successively to amplify flanking regions from self-ligated metagenome DNA. DNA walking was performed by using the DNA Walking SpeedUp kit (Seegene, Seoul, South Korea) according to manufacturer's protocol. PCR products from both inverse PCR and DNA walking were inserted into a vector using the TOPO TA Cloning Kit (Invitrogen, Carlsbad, CA, USA), and plasmids were isolated for sequencing analysis. Restriction endonucleases were purchased from Invitrogen or New England BioLabs (Ipswich, MA, USA); Taq polymerases were purchased from Invitrogen or Promega (Madison, WI, USA).

Two sets of plasmids were constructed for protein expression: one with full-length ORF and the other without the putative N-terminal signal peptide. Coding sequences were PCR amplified from metagenome DNA or fosmid DNA using primers that were designed according to each candidate's sequence, and were subsequently cloned into the pET28a vector (Novagen). Each plasmid was confirmed by DNA sequencing and introduced into *E. coli *host ER2566 (New England BioLabs) for protein expression. Descriptions of selected glycoside hydrolase candidates that were cloned in this study and their Genbank accession numbers are listed in Table 1 and 2.

### Protein expression and purification

For batch culture of *E. coli *bearing plasmid, cells were incubated in LB medium with 50 μg/ml kanamycin at 37°C until OD_600 nm _= 0.5-0.6. The culture was induced with isopropyl-β-D-thiogalactopyranoside (IPTG; 0.4 mM final concentration) at 18°C for 4 h. The cells were harvested by centrifugation, resuspended in 1/20 culture volume of lysis-equilibration-wash (LEW) buffer (50 mM sodium phosphate, pH 8.0, 300 mM NaCl, 10 mM 2*-*mercaptoethanol, 10% Triton X-100), and disrupted by sonication. The cell lysates were centrifuged at 15,000 *g *for 15 min and both the supernatant and the pellet were examined with SDS-PAGE. PrepEase His-tagged High Yield Purification Resin (USB, Cleveland, OH, USA) was added into the supernatant and gently mixed at 4°C for 1 h. After binding, the resin was pelleted and washed twice with 10 resin volumes of LEW buffer, and was subsequently eluted with elution buffer (LEW buffer plus 250 mM imidazole). Eluted proteins were examined with SDS-PAGE, dialyzed, and then concentrated for further enzymatic assays.

### Enzyme activity assays

For initial testing, *E. coli *strains bearing candidate genes on plasmids were cultured as described in previous section. Cells were harvested and resuspended in 50 mM sodium phosphate (pH 8.0) buffer with 100 mM NaCl. After sonication, the whole cell lysate was tested for substrate specificity. In a repeat experiment, CelLytic B lysis reagent (Sigma-Aldrich) was also used for cell lysis and supernatant was used for enzyme activity examination. Candidate clones and proteins were tested for enzyme activities using following substrates: *p*-nitrophenyl β-D-cellobioside, *p*-nitrophenyl β-D-glucopyranoside, *p*-nitrophenyl β-D-lactopyranoside, *p*-nitrophenyl β-D-galactopyranoside, *p*-nitrophenyl β-D-xylopyranoside, and *p*-nitrophenyl α-L-arabinofuranoside (all purchased from Sigma-Aldrich). Cell lysates or proteins were tested at 37°C, in 50 mM sodium phosphate buffer (pH 8.0) containing 100 mM NaCl and 0.5 mM substrate. After incubation for appropriate amount of time, the reactions were stopped by adding a quarter volume of 1 M Na_2_CO_3 _solution, and the hydrolysis product *p*-nitrophenol was measured by absorbance at 405 nm. A pure *p*-nitrophenol (Sigma-Aldrich) was used for producing a standard curve. The assay was performed with biological duplicates for each clone on every substrate.

To determine the pH optimum of candidate proteins we added 1 μL cell lysate, or purified proteins (final concentration 6.5 μg/ml), to solution of *p*-nitrophenyl substrates (final concentration 0.625 mM) in the following buffer range pH 4 to 8.5 (see Table 3). The total volume for the reaction was 200 μL. Reactions were conducted for 30 min at 30°C and quenched by the addition of 50 μL 1 M NaCO_3_. Absorbance was read at 405 nm to determine the extent of conversion. Data were normalized for 100% response at the maximum conversion.

**Table 3:** Buffers for the pH optimum assay

pH	Buffer
pH 4.5	50 mM Citrate, 100 mM NaCl
pH 5	50 mM Acetate, 100 mM NaCl
pH 5.5	50 mM Acetate, 100 mM NaCl
pH 6	50 mM Phosphate, 100 mM NaCl
pH 6.5	50 mM Phosphate, 100 mM NaCl
pH 7	50 mM Phosphate, 100 mM NaCl
pH 7.5	50 mM Phosphate, 100 mM NaCl
pH 8	50 mM Tris, 100 mM NaCl

To determine the temperature optimum of candidate proteins we added 1 μl cell lysate, or purified proteins (final concentration 6.5 μg/ml), to solution of *p*-nitrophenyl substrates (final concentration 0.625 mM) in a buffer that has the optimal pH range for the candidate. The total volume for the reaction was 200 μl. The temperature range used was from 25°C to 55°C in 5°C increments. Both the enzyme stock solution and the reaction mix were pre-equilibrated for 5 min at each tested temperature prior to mixing. Reactions were conducted for 10 min and quenched by the addition of 50 μl 1 M NaCO_3_. Absorbance was read at 405 nm to determine the extent of conversion. Data were normalized for 100% response at the maximum conversion.

### Enzyme tolerance of ionic liquids

To determine the effect of ionic liquids to candidate proteins, 1 μl of purified protein was added into a 199 μl reaction mix including: 0%, 5%, 10%, 15%, or 20% of ionic liquid; 0.625 mM (final concentration) of *p*-nitrophenyl substrates in buffers that has the optimal pH range for individual candidates. For control reactions, 1 μl of purified protein was replaced with 1 μl of buffer. Reactions were conducted under the optimal temperature of individual candidates for 12 h. After observation for signs of protein denaturation and precipitation, reactions were quenched by the addition of 50 μl 1 M NaCO_3_. Absorbance was read at 405 nm to determine the extent of conversion. Data were normalized for 100% response at the absence of ionic liquid.

## Competing interests

PB, CD, and DAM are employed by Lucigen Corp., a company that manufactures cloning and expression products. The remaining authors declare that they have no competing interests.

## Authors' contributions

L-LL participated in the design of experiments, coordination of this study, preformed most of experiments including the selection of candidate genes, all the gene cloning, full-length ORF identification (inverse PCR, DNA walking, and blast searches), SNP studies (sequence analyses and SNP identification, SNP clones construction, examine protein expression and solubility for each clone), sequencing, blast searching, and identifying ORF of all positive clones from the metagenomic expression library, recombinant plasmids construction, protein expression and purification, enzyme activity/property assays and characterization, and wrote the manuscript. Y-BZ participated in the protein purification for SNP studies. MGB performed DNA sequencing for SNP and DNA walking studies. SMM performed the Blastx searches and identified putative glycoside hydrolases from metagenome data. RB preformed the pH and temperature assays for enzyme no. 6. WSA participated in the experiment design of enzyme assays. MEH participated in the conceiving, design, and coordination of this study. PB, CD, and DAM preformed the expression library construction and screening. ST isolated high molecular weight metagenomic DNA, participated in the conceiving, design, and coordination of this study. SGT managed the metagenome sequencing. DvdL participated in the conceiving, design, and coordination of this study, and is the principal investigator of this study. All authors read and approved the final manuscript.

## References

[B1] Himmel ME Ed Biomass Recalcitrance - Deconstructing the Plant Cell Wall for Bioenergy 2008 Chichester, UK: Wiley

[B2] Bayer EA Belaich J-P Shoham Y Lamed R The cellulosomes: multienzyme machines for degradation of plant cell wall polysaccharides Ann Rev Microbiol 2004 58 521 554 10.1146/annurev.micro.57.030502.091022 15487947

[B3] Bayer EA Shimon LJ Shoham Y Lamed R Cellulosomes - structure and ultrastructure J Struct Biol 1998 124 221 234 10.1006/jsbi.1998.4065 10049808

[B4] Saha BC Woodward J Eds Fuels and chemicals from biomass American Chemical Society Symposium Series (ACS, Washington, DC, 1997) 1997 Washington, DC: American Chemical Society 2 45

[B5] Feng Y Duan C Pang H Mo X Wu C Yu Y Cloning and identification of novel cellulase genes from uncultured microorganisms in rabbit cecum and characterization of the expressed cellulases Appl Microbiol Biotechnol 2007 75 319 328 10.1007/s00253-006-0820-9 17216439

[B6] Ferrer M Golyshina OV Chernikova TN Khachane AN Reyes-Duarte D Santos VA Strompl C Elborough K Jarvis G Neef A Yakimov MM Timmis KN Golyshin PN Novel hydrolase diversity retrieved from a metagenome library of bovine rumen microflora Environ Microbiol 2005 7 1966 2010 10.1111/j.1462-2920.2005.00920.x 16309396

[B7] Singh B Gautam SK Verma V Kumar M Singh B Metagenomics in animal gastrointestinal ecosystem: potential biotechnological prospects Anaerobe 2008 14 138 144 10.1016/j.anaerobe.2008.03.002 18457965

[B8] Flint HJ Bayer EA Rincon MT Lamed R White BA Polysaccharide utilization by gut bacteria: potential for new insights from genomic analysis Nat Rev Microbiol 2008 6 121 131 10.1038/nrmicro1817 18180751

[B9] Ohkuma M Symbioses of flagellates and prokaryotes in the gut of lower termites Trends Microbiol 2008 16 345 352 10.1016/j.tim.2008.04.004 18513972

[B10] Warnecke F Luginbühl P Ivanova N Ghassemian M Richardson TH Stege JT Cayouette M McHardy AC Djordjevic G Aboushadi N Sorek R Tringe SG Podar M Martin HG Kunin V Dalevi D Madejska J Kirton E Platt D Szeto E Salamov A Barry K Mikhailova N Kyrpides NC Matson EG Ottesen EA Zhang X Hernández M Murillo C Acosta LG Rigoutsos I Tamayo G Green BD Chang C Rubin EM Mathur EJ Robertson DE Hugenholtz P Leadbetter JR Metagenomic and functional analysis of hindgut microbiota of a wood-feeding higher termite Nature 2007 450 560 565 10.1038/nature06269 18033299

[B11] Scharf ME Tartar A Termite digestomes as sources for novel lignocellulases Biofuel Bioprod Biorefin 2008 2 540 552 10.1002/bbb.107

[B12] Zachary A Colwell RR Gut-associated microflora of *Limnoria tripunctata *in marine creosote-treated wood pilings Nature 1979 282 716 717 10.1038/282716a0

[B13] Allgaier M Reddy A Park JI Ivanova N D'haeseleer P Lowry S Sapra R Hazen TC Simmons BA VanderGheynst JS Hugenholtz P Targeted discovery of glycoside hydrolases from a switchgrass-adapted compost community PLoS ONE 2010 5 e8812 10.1371/journal.pone.0008812 20098679 PMC2809096

[B14] Schlüter A Bekel T Diaz NN Dondrup M Eichenlaub R Gartemann KH Krahn I Krause L Krömeke H Kruse O Mussgnug JH Neuweger H Niehaus K Pühler A Runte KJ Szczepanowski R Tauch A Tilker A Viehöver P Goesmann A The metagenome of a biogas-producing microbial community of a production-scale biogas plant fermenter analysed by the 454-pyrosequencing technology J Biotechnol 2008 136 77 90 10.1016/j.jbiotec.2008.05.008 18597880

[B15] Yu H Zeng G Huang H Xi X Wang R Huang D Huang G Li J Microbial community succession and lignocellulose degradation during agricultural waste composting Biodegradation 2007 18 793 802 10.1007/s10532-007-9108-8 17308882

[B16] Whitman WB Coleman DC Wiebe WJ Prokaryotes: the unseen majority Proc Natl Acad Sci USA 1998 95 6578 6583 10.1073/pnas.95.12.6578 9618454 PMC33863

[B17] Amann RJ Binder BL Chisholm SW Devereux R Stahl DA Combination of 16S rRNA targeted oligonucleotide probes with flow-cemetry for analysing mixed microbial populations Appl Environ Microbiol 1990 56 1910 1925 10.1128/aem.56.6.1919-1925.1990 PMC184531 2200342

[B18] Epstein SS Ed Uncultivated Microorganisms 2009 New York: Springer-Verlag

[B19] Daniel R The metagenomics of soil Nat Rev Microbiol 2005 3 470 478 10.1038/nrmicro1160 15931165

[B20] Lorenz P Eck J Metagenomics and industrial applications Nature 2005 3 510 516 10.1038/nrmicro1161 15931168

[B21] Li LL McCorkle S Monchy S Taghavi S van der Lelie D Bioprospecting metagenomes: glycosyl hydrolases for converting biomass Biotechnol Biofuel 2009 2 10 10.1186/1754-6834-2-10 PMC2694162 19450243

[B22] Tyson GW Chapman J Hugenholtz P Allen EE Ram RJ Richardson PM Solovyev VV Rubin EM Rokhsar DS Banfield JF Community structure and metabolism through reconstruction of microbial genomes from the environment Nature 2004 428 37 43 10.1038/nature02340 14961025

[B23] Fischer F Mutschler J Zufferey D Enzyme catalysis with small ionic liquid quantities J Ind Microbiol Biotechnol 2011 38 477 487 10.1007/s10295-010-0908-1 21107639

[B24] Himmel ME Ding SY Johnson DK Adney WS Nimlos MR Brady JW Foust TD Biomass recalcitrance: engineering plants and enzymes for biofuels production Science 2007 315 804 807 10.1126/science.1137016 17289988

[B25] Swatloski RP Spear SK Holbrey JD Rogers RD Dissolution of cellulose with ionic liquids J Am Chem Soc 2002 124 4974 4975 10.1021/ja025790m 11982358

[B26] Remsing RC Swatloski RP Rogers RD Moyna G Mechanism of cellulose dissolution in the ionic liquid 1-n-butyl-3-methylimidazolium chloride: a 13C and 35/37Cl NMR relaxation study on model systems Chem Commun (Camb) 2006 12 1271 1273 10.1039/b600586c 16538244

[B27] El Seoud OA Koschella A Fidale LC Dorn S Heinze T Applications of ionic liquids in carbohydrate chemistry: a window of opportunities Biomacromolecules 2007 8 2629 2647 10.1021/bm070062i 17691840

[B28] Turner MB Spear SK Huddleston JG Holbrey JD Rogers RD Ionic liquid salt-induced inactivation and unfolding of cellulase from *Trichoderma reesei* Green Chem 2003 5 447

[B29] Schmidt TM DeLong EF Pace NR Analysis of a marine picoplankton community by 16S rRNA gene cloning and sequencing J Bacteriol 1991 173 4371 4378 2066334 10.1128/jb.173.14.4371-4378.1991 PMC208098

[B30] Himmel ME Xu Q Luo Y Ding S-Y Lamed R Bayer EA Microbial enzyme systems for biomass conversion: emerging paradigms Biofuels 2010 1 323 341 10.4155/bfs.09.25

